# Schwefelkohlenstoff – Bestimmung von Schwefelkohlenstoff in der Luft am Arbeitsplatz mittels Headspace-Gaschromatographie (Headspace-GC-FPD): Luftanalysen-Methode

**DOI:** 10.34865/am7515d9_4or

**Published:** 2024-12-23

**Authors:** Andreas Grill, Claus-Peter Maschmeier, Ralph Hebisch, Andrea Hartwig, MAK Commission

**Affiliations:** 1 Kelheim Fibres GmbH Regensburger Str. 109 93309 Kelheim Deutschland; 2 Land Sachsenanhalt Gebrüder-Bethmann-Str. 18 06862 Dessau-Roßlau Deutschland; 3 Bundesanstalt für Arbeitsschutz und Arbeitsmedizin (BAuA) Friedrich-Henkel-Weg 1–25 44149 Dortmund Deutschland; 4 Institut für Angewandte Biowissenschaften. Abteilung Lebensmittelchemie und Toxikologie. Karlsruher Institut für Technologie (KIT) Adenauerring 20a, Geb. 50.41 76131 Karlsruhe Deutschland; 5 Ständige Senatskommission zur Prüfung gesundheitsschädlicher Arbeitsstoffe. Deutsche Forschungsgemeinschaft, Kennedyallee 40, 53175 Bonn, Deutschland. Weitere Informationen: Ständige Senatskommission zur Prüfung gesundheitsschädlicher Arbeitsstoffe | DFG

**Keywords:** Schwefelkohlenstoff, Luftanalysen, Analysenmethode, Arbeitsplatzmessung, Gefahrstoff, Headspace-Gaschromatographie, flammenphotometrische Detektion, Headspace-GC-FPD, Aktivkohle, Flüssigdesorption

## Abstract

The working group “Air Analyses” of the German Senate Commission for the Investigation of Health Hazards of Chemical Compounds in the Work Area (MAK Commission) developed and verified the presented analytical method. It is used to the determine the levels of carbon disulfide [75-15-0] that occur in the workplace air. The method covers concentrations in the range from one tenth to twice the current German Occupational Exposure Limit Value (OELV) of 30 mg/m^3^. It is also suitable for monitoring compliance with the MAK value of 16 mg/m^3^ and the short-term exposure limit (STEL; excursion factor 2). Samples are collected by drawing a defined volume of air through a sampling tube filled with activated carbon using a flow regulated pump at a maximal volumetric flow rate of 0.333 l/min. Exposure during the shift is measured with a sampling period of 2 hours (up to 8 hours, depending on the volumetric flow) and the short-term exposure with a period of 15 minutes. The carbon disulfide adsorbed to the activated carbon is extracted with toluene and analysed by headspace gas chromatography with flame photometric detection. The quantitative determination is based on multiple-point calibrations with an internal standard. A relative limit of quantifiation (LOQ) of 0.5 mg/m^3^ is obtained for an air sample volume of 40 litres and a usage volume of 18 ml. As the relative LOQ for a sample volume of 5 litres is below 5 mg/m^3^, the STEL can also be measured. The recovery, which has to be considered for the calculation of the results, is approx. 70% and the expanded uncertainty is below 22% for a sampling period of 2 hours and below 23% for a period of 15 minutes.

**Table:** 

**Methodennummer**	3
**Anwendbarkeit**	Luftanalyse
**Analyt. Messprinzip**	Headspace-Gaschromatographie mit flammenphotometrischer Detektion (Headspace-GC-FPD)

## 1 Kenndaten des Verfahrens

**Table:** 

**Präzision:**	Variationskoeffizient:	*s* = 2,4 bis 6,7 %
	Erweiterte Messunsicherheit:	*U* = 21,2 bis 21,8 %
	in einem Bereich von 3,0–45 mg/m^3^ und n = 5	
**Bestimmungsgrenze:**	Absolut:	0,5 mg/l
	Relativ:	0,5 mg/m^3^ bei einem Probeluftvolumen von 40 l und einem Anwendungsvolumen von 18 ml 4 mg/m^3^ bei einem Probeluftvolumen von 5 l für Kurzzeitmessungen und einem Anwendungsvolumen von 18 ml
**Wiederfindung:**	*η* = ca. 70 % (wird in das Ergebnis eingerechnet, muss bestimmt werden)	
**Probenahmeempfehlung:**	Probenahmedauer:	2 h bis 8 h
	Probeluftvolumen:	40 l
	Volumenstrom:	0,333 bis 0,083 l/min
	Für Kurzzeitmessungen:	15 min; 0,333 l/min

## 2 Stoffbeschreibung

### Schwefelkohlenstoff [75-15-0]

Schwefelkohlenstoff (Formel: S=C=S, CS_2_, auch Kohlenstoffdisulfid genannt) ist eine farblose, stark lichtbrechende Flüssigkeit. In reinem Zustand riecht Schwefelkohlenstoff angenehm wie Ether, durch Verunreinigungen ist dieser etherische Geruch meist nicht mehr wahrnehmbar. Geringe Mengen von anderen Schwefelverbindungen sind die Ursache für einen teilweise unangenehmen Geruch. Schwefelkohlenstoff besitzt einen sehr niedrigen Flammpunkt und bildet in Konzentrationen von 0,6–60 Vol% explosive Dampf-Luft-Gemische (RÖMPP-Redaktion und Sitzmann 2009).

Schwefelkohlenstoff wird in großen Mengen zur Herstellung von Cellulosefasern aus Zellstoff eingesetzt, wobei der Zellstoff zuerst mit Natronlauge zu Alkalicellulose umgesetzt und diese nach dem oxidativen Abbau mit Schwefelkohlenstoff zu dem in Natronlauge löslichen Xanthogenat verarbeitet wird. Die so entstandene Celluloselösung, auch Viskose genannt, wird in schwefelsauren Spinnbädern zu Regeneratcellulose versponnen. Darüber hinaus wird es im Weinbau gegen die Reblaus, als Reagenz in Synthesen und in der IR-Spektroskopie als Lösungsmittel eingesetzt. Es ist außerdem ein gutes Lösungsmittel für Fette, Harze, Gummi und Kautschuk (IFA 2024; RÖMPP-Redaktion und Sitzmann 2009).

Der Arbeitsplatzgrenzwert (AGW) für Schwefelkohlenstoff beträgt 30 mg/m^3^ (10 ml/m^3^), der Kurzzeitwert ist der Spitzenbegrenzungs-Kategorie II mit dem Überschreitungsfaktor 2 zugeordnet (AGS 2024). In der MAK- und BAT-Werte-Liste hat Schwefelkohlenstoff einen MAK-Wert von 16 mg/m^3^ in etwa halber Höhe des AGW (DFG 2024), ebenfalls mit der Spitzenbegrenzungs-Kategorie II und Überschreitungsfaktor 2. Stoffdaten zu Schwefelkohlenstoff können der Tabelle 1 entnommen werden.

**Tab.1:** Stoffdaten zu Schwefelkohlenstoff (IFA 2024)

Name	Schwefelkohlenstoff
CAS-Nr.	75-15-0
Molmasse [g/mol]	76,14
Aggregatzustand bei 20 °C	flüssig
Dichte bei 20 °C [g/cm^3^]	1,26
Dampfdruck bei 20 °C [hPa]	395
Schmelzpunkt [°C]	–112
Siedepunkt bei 1013 hPa [°C]	46
Flammpunkt [°C]	< –20
Beurteilungsmaßstäbe	
AGW, Deutschland (AGS 2024)	30 mg/m^3^
MAK-Wert, Deutschland (DFG 2024)	16 mg/m^3^

## 3 Grundlage des Verfahrens

Mit diesem Analysenverfahren kann Schwefelkohlenstoff in der Luft am Arbeitsplatz in einem Konzentrationsbereich vom 0,1- bis zum 2-Fachen des derzeit gültigen AGW von 30 mg/m^3^ bestimmt werden (AGS 2024). Auch die Einhaltung der Spitzenbegrenzung mit einem Überschreitungsfaktor von 2 kann überprüft werden (AGS 2024; DIN 2021 a). Die Bestimmungsgrenze des Verfahrens ist zudem ausreichend niedrig, um den MAK-Wert von 16 mg/m^3^ als Beurteilungsmaßstab heranzuziehen.

Zur Probenahme wird mit Hilfe einer Probenahmepumpe ein definiertes Luftvolumen aus dem Atembereich durch ein Aktivkohleröhrchen (Typ B/G) gesaugt. Nach Flüssigdesorption mit Toluol und Headspace-Gaschromatographie erfolgt eine flammenphotometrische Detektion (Headspace-GC-FPD). Die quantitative Auswertung erfolgt anhand einer Mehrpunktkalibrierung mit internem Standard (ISTD, Thiophen).

## 4 Geräte, Chemikalien und Lösungen

### 4.1 Geräte

Für die Probenahme:

- Probenahmepumpe für personengetragene und stationäre Probenahme, geeignet für einen Volumenstrom zwischen 0,083 l/min und 0,333 l/min (z. B. SG350ex, DEHA Haan & Wittmer GmbH, 71296 Heimsheim)
- Aktivkohleröhrchen Typ B/G (z. B. Fa. Drägerwerk AG & Co. KGaA, 23558 Lübeck)
- Röhrchenhalter in geeigneter Größe (z. B. Art.-Nr. DH520060, Fa. DEHA Haan & Wittmer GmbH, 71296 Heimsheim)
- Silikonschlauch und Olive zur Verbindung von Pumpe und Röhrchenhalter
- Durchflussmesser (z. B. Gilian Gilibrator 3, Fa. Sensidyne LP, St. Petersburg, FL, USA, Vertrieb durch Fa. DEHA Haan & Wittmer GmbH, 71296 Heimsheim)

Für die Probenaufbereitung und analytische Bestimmung:

- Headspace-Gläschen (z. B. Rollrandflasche ND20, Fa. Th. Geyer GmbH & Co. KG, 71272 Renningen)
- Verschlussdeckel (Aluminium/Silikon) (z. B. Bördelkappe ND20, Fa. Th. Geyer GmbH & Co. KG, 71272 Renningen)
- Glasschneider (z. B. Fa. Carl Friedrich Usbeck KG, 42477 Radevormwald)
- Messkolben aus Glas (25 bis 200 ml) mit Glasstopfen (z. B. Blaubrand, Fa. Brand GmbH und Co + KG, 97877 Wertheim)
- Vollpipetten aus Glas (1 bis 10 ml) (z. B. Blaubrand, Fa. Brand GmbH und Co + KG, 97877 Wertheim)
- Einmalspritzen, 20 ml, aus Polyethylen mit passenden Kanülen
- Analysenwaage (z. B. A200S, Fa. Sartorius AG, 37079 Göttingen)
- Gaschromatograph mit CAP-Inlet mit deaktiviertem Liner und flammenphotometrischem Detektor, sowie Steu­erungs- und Auswertesoftware (z. B. Clarus 590, PerkinElmer LAS Germany GmbH, 63110 Rodgau)
- Headspace-Probengeber (z. B. Turbomatrix 40, PerkinElmer LAS Germany GmbH, 63110 Rodgau)
- Trennsäule GS-Q 30 m × 0,53 mm (z. B. J&W, Fa. Agilent Technologies Deutschland GmbH, 76337 Waldbronn)

### 4.2 Chemikalien

- Toluol, ≥ 99,9 % (z. B. Fa. Merck KGaA, 64271 Darmstadt)
- Schwefelkohlenstoff, ≥ 99,90 % (z. B. Fa. Honeywell International Inc., Morristown, NJ, USA). Es werden zwei unab­hängige Chargen für die Herstellung von Kalibrier- und Kontrollstandards benötigt.
- Thiophen, zur Synthese, 99,9 % (z. B. Fa. Merck KGaA, 64293 Darmstadt)
- Helium 5.0 (Trägergas) (z. B. Fa. Air Liquide Deutschland GmbH, 40476 Düsseldorf)
- Wasserstoff 5.0 (z. B. Fa. Air Liquide Deutschland GmbH, 40476 Düsseldorf)
- Synthetische Luft 5.0 (kohlenwasserstofffrei) (z. B. Fa. Air Liquide Deutschland GmbH, 40476 Düsseldorf)
- Prüfgas 30 mg CS_2_/m^3^ in Luft (z. B. Fa. Air Liquide Deutschland GmbH, 40476 Düsseldorf)

### 4.3 Lösungen 

Die folgenden Lösungen wurden mit den Chemikalien, welche in Abschnitt 4.2 aufgelistet sind, hergestellt, alle Lösungen sind im Kühlschrank aufzubewahren:

**Thiophen-Stammlösung:** (5 g Thiophen/l in Toluol)

0,50 g Thiophen werden eingewogen und in einen 100-ml-Messkolben überführt. Anschließend wird der Kolben mit Toluol bis zur Marke aufgefüllt und geschüttelt. Die Lösung kann bei Lagerung im Kühlschrank für ein Jahr verwendet werden.

**Thiophen-Standardlösung:** (250 mg Thiophen/l in Toluol)

In einen 200-ml-Messkolben, in dem ca. 100 ml Toluol vorgelegt wurden, werden 10 ml der Thiophen-Stammlösung zugegeben. Danach wird der Messkolben mit Toluol bis zur Marke aufgefüllt und geschüttelt. Die Lösung kann bei Lagerung im Kühlschrank für sechs Monate verwendet werden.

**Schwefelkohlenstoff-Stammlösung:** (5 g CS_2_/l in Toluol)

0,50 g Schwefelkohlenstoff werden eingewogen und in einen 100-ml-Messkolben überführt. Anschließend wird der Kolben mit Toluol bis zur Marke aufgefüllt und geschüttelt. Die Lösung kann bei Lagerung im Kühlschrank für ein Jahr verwendet werden.

Es wird eine zweite, analoge Stammlösung mit einer unabhängigen Charge Schwefelkohlenstoff für die Herstellung der Kontrollstandards angesetzt. 

**Kalibrierlösung:** (250 mg CS_2_/l in Toluol)

In einen 200-ml-Messkolben, in dem ca. 100 ml Toluol vorgelegt wurde, werden 10 ml der Schwefelkohlenstoff-Stammlösung zugegeben. Danach wird der Messkolben mit Toluol bis zur Marke aufgefüllt und geschüttelt. Die Lösung kann bei Lagerung im Kühlschrank für einen Monat verwendet werden.

**Kontrolllösung:** (250 mg CS_2_/l in Toluol)

In einen 100-ml-Messkolben, in dem ca. 50 ml Toluol vorgelegt wurden, werden 5 ml der Schwefelkohlenstoff-Stammlösung für die Kontrolle zugegeben. Danach wird der Messkolben mit Toluol bis zur Marke aufgefüllt und geschüttelt. Die Lösung kann bei Lagerung im Kühlschrank für einen Monat verwendet werden.

**Blindwertlösung:** (25 mg Thiophen/l in Toluol)

In einen 50-ml-Messkolben, in dem ca. 25 ml Toluol vorgelegt wurden, werden 5 ml der Thiophen-Standardlösung zugegeben. Danach wird der Messkolben mit Toluol bis zur Marke aufgefüllt und geschüttelt. Die Blindwertlösung wird arbeitstäglich frisch hergestellt.

### 4.4 Kalibrier- und Kontrollstandards

Die Kalibrierstandards werden durch Verdünnungen der Kalibrierlösung in Toluol entsprechend der Tabelle 2 hergestellt. Dazu werden in zehn 100-ml-Messkolben je ca. 50 ml Toluol vorgelegt und die in Tabelle 2 aufgeführten Volumina mittels Vollpipetten zugegeben. Anschließend werden die Messkolben jeweils mit Toluol bis zur Marke aufgefüllt und gründlich geschüttelt. 

Es wird jeweils die Aktivkohle der Sammelschicht eines Typ B/G-Röhrchens in ein Rollrandfläschchen gegeben und jeweils mit 20 ml der jeweiligen Kalibrierstandards überschichtet. Die Gefäße werden verschlossen und eine Stunde bei Raumtemperatur stehen gelassen.

Je 5 ml dieser Lösungen werden jeweils mit Einmalspritzen in 25-ml-Messkolben überführt und je 2,5 ml Thiophen-Standardlösung hinzugegeben. Die Messkolben werden mit Toluol bis zur Marke aufgefüllt und gründlich geschüttelt. Jeweils 5 ml dieser Kalibrierproben werden in Headspace-Gläschen gegeben und unter den in Abschnitt 6 genannten Arbeitsbedingungen analysiert.

**Tab.2:** Herstellung und Konzentrationen der Kalibrierstandards und -proben

**Kalibrierstandard**	**1**	**2**	**3**	**4**	**5**	**6**	**7**	**8**	**9**	**10**
V (Kalibrierlösung) [ml]	1	2	3	4	5	6	7	8	9	10
*c* (Kalibrierstandard) [mg/l]	2,50	5,00	7,50	10,0	12,5	15,0	17,5	20,0	22,5	25,0
*c* (Kalibrierprobe) [mg/l]	0,500	1,00	1,50	2,00	2,50	3,00	3,50	4,00	4,50	5,00
ρ (CS_2_) [mg/m^3^]^a)^	6,25	12,5	18,8	25,0	31,3	37,5	43,8	50,0	56,3	62,5
ρ (CS_2_) [mg/m^3^]^b)^	1,25	2,50	3,75	5,00	6,25	7,50	8,75	10,0	11,3	12,5
ρ (CS_2_) [mg/m^3^]^c)^	0,625	1,25	1,88	2,50	3,13	3,75	4,38	5,00	5,63	6,25

a) für ein Probenahmevolumen von 40 l (120 min mit 0,333 l/min) und ein Anwendungsvolumen von 1 ml

b) für ein Probenahmevolumen von 40 l (120 min mit 0,333 l/min) und ein Anwendungsvolumen von 5 ml

c) für ein Probenahmevolumen von 40 l (120 min mit 0,333 l/min) und ein Anwendungsvolumen von 10 ml

Der kalibrierte Bereich entspricht für ein Probenahmevolumen von 40 l (120 min, 0,333 l/min) und eine Anwendung der Desorptionslösung von 5 ml 1,3–13 mg/m^3^. Entsprechend verringert sich der Kalibrierbereich für eine Anwendung von 10 ml auf 0,63–6,3 mg/m^3.^ Genauso erhöht sich der Kalibrierbereich für eine Anwendung von 1 ml auf 6,3–63 mg/m^3^ (siehe Tabelle 2).

Durch die Variation der Anwendung kann der Kalibrierbereich je nach erwartetem Gehalt genutzt werden. Mit entsprechenden Verdünnungen kann man auch Gehalte analysieren, die nahe dem AGW oder sogar darüber liegen. Der geforderte Bereich von einem Zehntel bis zum Doppelten des derzeit gültigen AGW kann somit abgedeckt werden.

Arbeitstäglich werden frische Kontrollstandards durch Verdünnungen der Kontrolllösung in Toluol entsprechend der Tabelle 3 hergestellt. Dazu werden in zwei 50-ml-Messkolben je ca. 25 ml Toluol vorgelegt und die in Tabelle 3 aufgeführten Volumina mittels Vollpipetten zugegeben. Anschließend werden die Messkolben jeweils mit Toluol bis zur Marke aufgefüllt und gründlich geschüttelt.

Die Aktivkohle der Sammelschicht (größere Aktivkohleschicht) eines Typ B/G-Röhrchens wird in ein Rollrandfläschchen gegeben und jeweils mit 20 ml des jeweiligen Kontrollstandards überschichtet. Die Gefäße werden verschlossen und eine Stunde bei Raumtemperatur stehen gelassen.

Je 5 ml dieser Lösungen werden jeweils mit Einmalspritzen in 25-ml-Messkolben überführt und je 2,5 ml Thiophen-Standardlösung hinzugegeben. Die Messkolben werden mit Toluol bis zur Marke aufgefüllt und gründlich geschüttelt. Jeweils 5 ml dieser Kontrollprobe werden in Headspace-Gläschen gegeben und unter den in Abschnitt 6 genannten Arbeitsbedingungen analysiert.

**Tab.3:** Herstellung und Konzentrationen der Kontrollstandards und -proben

Kontrollstandard	1	2
V (Kontrolllösung) [ml]	1	4
*c *(Kontrollstandard) [mg/l]	5,00	20,0
*c *(Kontrollprobe) [mg/l]	1,00	4,00
ρ (CS_2_) [mg/m^3^]^a)^	12,5	50,0
ρ (CS_2_) [mg/m^3^]^b)^	2,50	10,0
ρ (CS_2_) [mg/m^3^]^c)^	1,25	5,00

a) für ein Probenahmevolumen von 40 l (120 min mit 0,333 l/min) und ein Anwendungsvolumen von 1 ml

b) für ein Probenahmevolumen von 40 l (120 min mit 0,333 l/min) und ein Anwendungsvolumen von 5 ml

c) für ein Probenahmevolumen von 40 l (120 min mit 0,333 l/min) und ein Anwendungsvolumen von 10 ml

## 5 Probenahme und Probenaufbereitung

### 5.1 Probenahme

**Abb.1:**
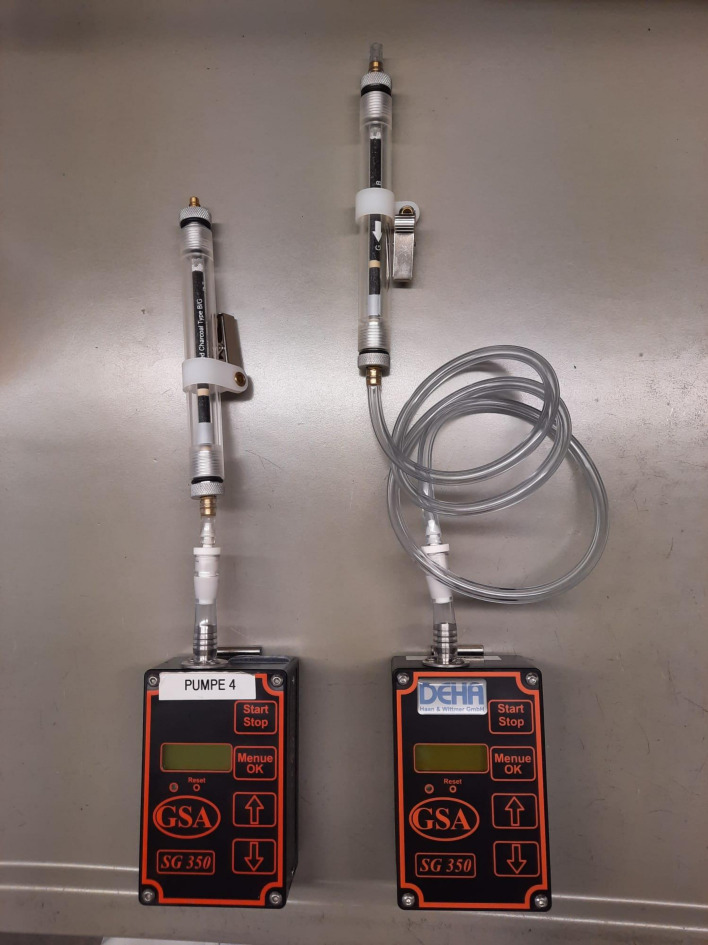
Aufbau der Probenträger, links für stationäre Messungen, rechts für personengetragene Messungen, zusammengesetzt aus Sammelröhrchen (Typ B/G) im Röhrchenhalter, Verbindungsschlauch und Probenahmepumpe (SG350ex)

Das Aktivkohleröhrchen wird unmittelbar vor der Probenahme mit einem Glasschneider geöffnet, dicht in einen Röhrchenhalter eingespannt und dieser mit der Pumpe über ein Schlauchstück in geeigneter Länge (personengetragen oder stationär) verbunden (siehe Abbildung 1, links stationär, rechts personengetragen). Dabei wird auf die Flussrichtung der Luft geachtet und die größere Aktivkohleschicht als Sammelschicht verwendet (Typ G).

Mithilfe eines repräsentativen Probenträgers der gleichen Herstellungscharge wird der Volumenstrom auf z. B. 5 l/h (0,083 l/min) oder 20 l/h (0,333 l/min) je nach geplanter Messdauer eingestellt. Die empfohlene Probenahmedauer ­beträgt für die Bestimmung des Schichtmittelwertes mindestens 120 Minuten. Das empfohlene Probenahmevolumen liegt zwischen 5 und 60 l.

Ist am Ende der Probenahme die Abweichung vom anfangs eingestellten Volumenstrom größer als ± 5 %, wird empfohlen, die Probe zu verwerfen (DIN 2023).

### 5.2 Probenaufbereitung

Die Probenträger werden spätestens 7 Tage nach der Probenahme bearbeitet.

Die Aktivkohle der Sammel- und Kontrollschicht des Typ B/G-Röhrchens wird getrennt in je ein Rollrandfläschchen gegeb­en und jeweils mit 20 ml Toluol überschichtet. Die Gefäße werden verschlossen und eine Stunde bei Raum­temperatur stehen gelassen.

Die Desorptionslösung (Extrakt der Sammelschicht) wird mittels einer Einmalspritze aufgenommen und ein definiertes Anwendungsvolumen (z. B. 6 ml) in einen 25-ml-Messkolben gegeben. Anschließend werden 2,5 ml der Thiophen-Standardlösung (siehe Abschnitt 4.3) mittels einer Vollpipette hinzugegeben und der Messkolben bis zur Marke mit Toluol aufgefüllt. 5 ml dieser Lösung werden in Headspace-Gläschen gegeben und unter den in Abschnitt 6 genannten Arbeitsbedingungen analysiert.

Um den MAK-Wert (16 mg/m^3^) abzudecken, empfiehlt sich bei einem Probeluftvolumen von 40 l eine Anwendung von 6 ml Desorptionslösung (Arbeitsbereich ca. 1,5–15 mg/m^3^). Um den AGW von 30 mg/m^3^ abzudecken, empfiehlt sich bei 40 l Probeluftvolumen eine Anwendung von 3 ml Desorptionslösung (Arbeitsbereich ca. 3,1–31 mg/m^3^).

Aus dem Extrakt der Kontrollschicht werden mittels einer 20-ml-Einmalspritze 5 ml in ein Headspace-Gläschen gege­ben und ohne ISTD analysiert. Sollte in der Kontrollschicht Schwefelkohlenstoff detektiert werden, ist eine Probe unter Zusatz des ISTDs analog zu einer Sammelschicht erneut zu analysieren.

## 6 Instrumentelle Arbeitsbedingungen

Die analytische Bestimmung erfolgt unter den aufgelisteten Bedingungen mit einem Gaschromatographie-System aus Headspace-Probengeber mit Platinnadel, CAP-Inlet mit deaktiviertem Liner und einem flammenphotometrischen Detektor.

**Table:** 

**Headspace-Probengeber:**	
**Gerät:**	Turbomatrix 40, PerkinElmer LAS Germany GmbH
**Injektionsmodus:**	Zeit
**Ofentemperatur:**	90 °C
**Nadeltemperatur:**	100 °C
**Transferlinetemperatur:**	110 °C
**Transferlinedruck:**	160 kPa
**Thermostatisierzeit:**	20 min
**Druckaufbauzeit:**	3 min
**Injektionszeit:**	0,05 min
**Verweilzeit:**	0,2 min
**GC-Zykluszeit:**	10 min
**Bedienungsmodus:**	konstant

**Gaschromatograph:**	
**Gerät:**	Clarus 590, PerkinElmer LAS Germany GmbH
**Säule:**	J&W, GS-Q 30 m × 0,53 mm
**Ofentemperatur:**	150 °C (isotherm)
**Trägergas:**	Helium
**Trägergasdruck:**	70 kPa (druckgesteuert)
**Injektortemperatur:**	250 °C
**Detektor:**	flammenphotometrischer Detektor
**Detektortemperatur:**	320 °C
**Range:**	1
**Attenuation:**	0

Unter den angegebenen Bedingungen hat Schwefelkohlenstoff eine Retentionszeit von ca. 1,6 Minuten.

## 7 Analytische Bestimmung

Zur analytischen Bestimmung werden die nach Abschnitt 5.2 aufbereiteten Proben, mit Hilfe des Headspace-Probengebers in den Gaschromatographen injiziert und unter den in Abschnitt 6 angegebenen Bedingungen in einer Doppelbestimmung analysiert. Dazu werden zwei Headspace-Gläschen präpariert und getrennt analysiert. Liegen die ermittelten Konzentrationen oberhalb des Kalibrierbereiches, so sind geeignete Verdünnungen herzustellen und diese nochmals zu analysieren. Des Weiteren wird ein Reagenzienblindwert („Lab Blank“) analog den Analysenproben analysiert. Dazu werden 5 ml der Blindwertlösung (siehe Abschnitt 4.3) in ein Headspace-Gläschen überführt und unter den in Abschnitt 6 aufgeführten Bedingungen analysiert.

## 8 Kalibrierung

Zur Erstellung der Kalibrierfunktion werden die unter Abschnitt 4.4 beschriebenen Kalibrierproben entsprechend den Abschnitten 6 und 7 analysiert und die ermittelten Quotienten der Peakflächen von Schwefelkohlenstoff und ISTD gegen die jeweiligen Konzentrationen der Kalibrierprobe aufgetragen.

Die Kalibrierfunktion ist im untersuchten Konzentrationsbereich quadratisch und sollte in der Routineanalytik regelmäßig überprüft werden. Dazu sind bei jeder Analysenreihe zwei Kontrollstandards bekannter Konzentration zu analysieren (siehe Abschnitt 4.4).

Haben sich die analytischen Bedingungen geändert oder die Qualitätskontrolle gibt Anlass dazu, ist eine neue Kalibrierung zu erstellen. 

## 9 Berechnung des Analysenergebnisses

Anhand der Peakflächen wird aus der Kalibrierfunktion die Konzentration der Probenlösung bestimmt. Mit Zuhilfenahme des Desorptionsvolumens, des Volumens der Anwendung und dem Volumen der Probelösung wird die zugehörige Masse *X* in mg je Probe ermittelt. 


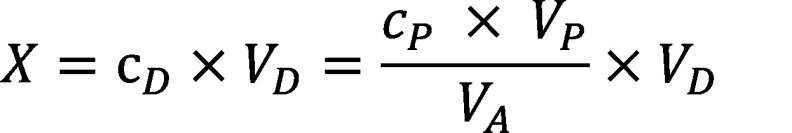
(1)

Es bedeuten: 

**Table:** 

*X*	Masse der Substanz pro Probenträger in mg
*c_D_*	Konzentration der Substanz in der Desorptionslösung in mg/l
*V_D_*	Volumen der Desorptionslösung (hier 0,02 l)
*V_P_*	Volumen der Probelösung (hier 0,025 l)
*V_A_*	Volumen der Anwendung in Liter
*c_P_*	Konzentration der Substanz in der Probenlösung in mg/l

Die Massenkonzentration (ρ) errechnet sich nach Gleichung 2. Das berechnete Analysenergebnis ist mit der Wieder­findung *ƞ *von ca. 70 % (siehe Abschnitt 10.2) zu korrigieren. 


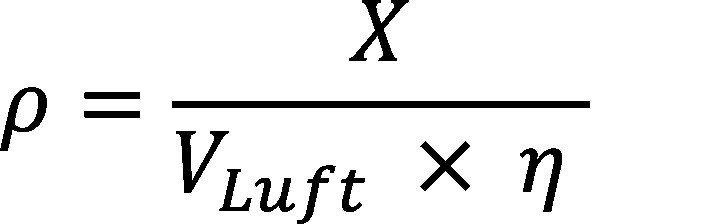
(2)

Zur Umrechnung auf 20 °C und 1013 hPa gilt Gleichung 3:


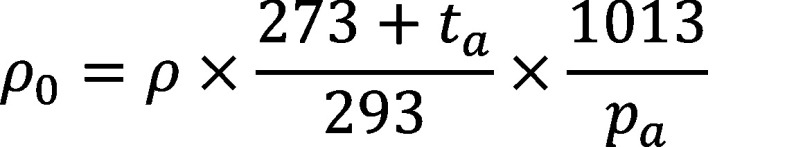
(3)

Es bedeuten: 

**Table:** 

*ρ*	Massenkonzentration der Substanz in der Luftprobe in mg/m^3^ bezogen auf *t_a_* und *p_a_*
*ρ_0_*	Massenkonzentration der Substanz in mg/m^3^ bezogen auf 20 °C und 1013 hPa
*V_Luft_*	Probeluftvolumen in m^3^ (ermittelt aus Volumenstrom und Probenahmedauer)
*ƞ*	Wiederfindung (hier ca. 70 %)
*t_a_*	Temperatur während der Probenahme in °C
*p_a_*	Luftdruck während der Probenahme in hPa

## 10 Beurteilung des Verfahrens

Die Kenndaten der Methode wurden gemäß DIN EN 482 (DIN 2021 a), DIN EN ISO 22065 (DIN 2021 b) und DIN 32645 (DIN 2008) ermittelt. Es wurde eine vollständige Validierung der Methode durchgeführt. Die Leistungsfähigkeit des Verfahrens wurde anhand von Probenträgern ermittelt, die zuvor mit einem Prüfgas definierter Konzentration (29,58 mg/m^3^, siehe Abschnitt 4.2) belegt wurden. Dazu wurden die Probenträger durch einen Teflonschlauch bei Raumtemperatur (ca. 25 °C) mit einem vorgeschalteten Rotameter bei einem Volumenstrom von ca. 0,333 l/min für 12 min, 120 min oder 180 min belegt. Dies entspricht für eine zweistündige Probenahme 3 mg/m^3^, 30 mg/m^3^ bzw. 45 mg/m^3^, also etwa einem Zehntel des derzeit gültigen AGW, dem AGW und dem 1,5-Fachen des AGW. Bezogen auf den derzeit gültigen MAK-Wert entsprechen die beaufschlagten Mengen dem 0,2-Fachen, dem 2-Fachen bzw. dem 3-Fachen dessen für eine zweistündige Probenahme. Das exakte Volumen wurde durch einen nachgeschalteten Trommelgaszähler aufgezeichnet.

### 10.1 Wiederholpräzision

Die Wiederholpräzision wurde anhand von jeweils fünf mit Prüfgas (Sollkonzentration 29,58 mg/m^3^) beaufschlagten Aktivkohleröhrchen bestimmt. Die Aktivkohleröhrchen wurden wie in Abschnitt 10 beschrieben jeweils für 12 min, 120 min oder 180 min beaufschlagt. Die Probenträger wurden wie in Abschnitt 5.2 beschrieben aufbereitet und unter den in den Abschnitten 6 und 7 beschriebenen Arbeitsbedingungen analysiert. Die Variationskoeffizienten wurden jeweils mit 6,66 %, 4,02 % bzw. 2,39 % berechnet.

### 10.2 Wiederfindung

Die Wiederfindung wurde aus den Daten der Präzision anhand von jeweils fünf mit Prüfgas (Sollkonzentration 29,58 mg CS_2_/m^3^) beaufschlagten Probenträgern ermittelt. Die Wiederfindung ist einmal pro Aktivkohle-Charge zu bestimmen, da sie von dieser abhängen kann. Sie liegt für alle untersuchten Konzentrationen bei ca. 70 % und muss deshalb bei der Berechnung des Ergebnisses berücksichtigt werden. Für die drei Konzentrationen wurden 72,1 %, 76,3 % und 72,8 % Wiederfindung ermittelt. Umgerechnet auf eine Wiederfindung von 70 % für die Charge der Aktivkohle entspricht dies 103 %, 109 % bzw. 104 %. 

### 10.3 Erweiterte Messunsicherheit

Die erweiterte Messunsicherheit wurde unter Berücksichtigung aller relevanten Einflussgrößen nach DIN EN 482 und ISO 20581 (DIN 2021 a, 2016) abgeschätzt und mit Hilfe des IFA-Excel-Sheets (IFA o.J.) zur Berechnung der erweiterten Messunsicherheit berechnet. Die Ergebnisunsicherheit des Gesamtverfahrens und damit des Analysenergebnisses setzt sich im Wesentlichen aus den Unsicherheitsbeträgen bei der Probenahme (z. B. Probeluftvolumen) und der analytischen Bearbeitung (z. B. vollständige Desorption, Streuung der Kalibrierfunktion, Schwankung der Wiederfindungen und der Reproduzierbarkeit) zusammen. Die kombinierte analytische Messunsicherheit beträgt maximal 11 %, die erwei­terte Messunsicherheit mit dem Erweiterungsfaktor k = 2 liegt für das Gesamtverfahren zwischen 21,2 und 21,8 %.

### 10.4 Einfluss der Luftfeuchte

Für die Bestimmung des Einflusses der Luftfeuchte wurden fünf Aktivkohleröhrchen mit Prüfgas (Sollkonzentration 29,58 mg CS_2_/m^3^) für 12 Minuten beaufschlagt. Durch diese wurde dann für zwei Stunden Luft mit einer relativen Luftfeuchte von 80 % und einem definierten Volumenstrom von 0,333 l/min gesaugt. Die Probenträger wurden wie unter Abschnitt 5.2 beschrieben aufgearbeitet und unter den in den Abschnitten 6 und 7 beschriebenen Bedingungen analysiert. Umgerechnet auf eine Wiederfindung von 70 % für die Charge der Aktivkohle wurden 111 % Schwefelkohlenstoff bestimmt. Bei höheren Luftfeuchten ist die Wiederfindung zu überprüfen und gegebenenfalls mit einem angepassten Korrekturfaktor zu berücksichtigen.

### 10.5 Bestimmungsgrenze

Die Ermittlung der Bestimmungsgrenze wurde aufgrund der quadratischen Kalibrierfunktion auf den ersten Kali­brierpunkt festgelegt und beträgt somit 0,5 mg/l absolut. Die relative Bestimmungsgrenze ist abhängig vom verwendeten Extraktionsvolumen und der Größe der Anwendung. Bei einem Extraktionsvolumen von 20 ml, einer Anwendung von 18 ml und einem Probeluftvolumen von 40 l ergibt sich eine relative Bestimmungsgrenze für den Schichtmittelwert von 0,5 mg/m^3^. Für die Bestimmung des Kurzzeitwertes mit einem Probeluftvolumen von 5 l resultiert ein Wert von 4 mg/m^3^.

### 10.6 Kapazität des Adsorbens

Zur Überprüfung der Kapazität des Sammelmediums wurden bei Raumtemperatur drei Aktivkohleröhrchen parallel mit Prüfgas einer Konzentration von 29,58 mg/m^3^ für drei Stunden mit einem Volumenstrom von 0,333 l/min beauf­schlagt. Dies entspricht etwa dem 1,5-Fachen des derzeit gültigen AGW bei einer zweistündigen Probenahme und 40 l Probenahmevolumen. In der Kontrollschicht wurden weniger als 5 % der gesamten wiedergefundenen Menge an Schwefelkohlenstoff bestimmt. Unter Berücksichtigung der experimentell bestimmten Wiederfindung von ca. 70 % betrug die Wiederfindung der Sammelschicht 98,5 %. Somit ist die Kapazität des Sammelmediums auch für eine Probenahmedauer von 3 Stunden bzw. für ein Probeluftvolumen von 60 l ausreichend.

Sollte das Probeluftvolumen 60 l übersteigen oder die erwarteten Luftkonzentrationen oberhalb von 45 mg/m^3^ liegen, muss ein zweites Aktivkohleröhrchen als Durchbruchssicherung verwendet werden.

### 10.7 Lagerfähigkeit

Zur Ermittlung der Lagerfähigkeit wurden jeweils sechs Aktivkohleröhrchen mit Prüfgas (29,58 mg/m^3^) mit einem Volumenstrom von 0,333 l/min für 12 oder 240 Minuten beladen. Dies entspricht für eine zweistündige Probenahme Luftkonzentrationen in Höhe von etwa einem Zehntel bzw. dem Doppelten des derzeit gültigen AGW. Jeweils drei Aktivkohleröhrchen einer Konzentration wurden direkt analysiert. Die jeweils anderen drei wurden nach sieben Tagen Lagerung bei 1–5 °C analysiert. Die Aufarbeitung erfolgte wie in Abschnitt 5.2 beschrieben. Nach der analytischen Bestimmung unter den in den Abschnitten 6 und 7 genannten Bedingungen und Einbeziehung der experimentell ermit­telten Wiederfindung wurden die in Tabelle 4 dargestellten Wiederfindungen ermittelt. Bei 1–5 °C sind die beaufschlagten Probenträger bis zu sieben Tage lagerfähig.

**Tab.4:** Wiederfindungen Lagerfähigkeit

Konzentration	Lagertage	Wiederfindung
0,1 AGW	0	95,2 %
2 AGW	0	102,3 %
0,1 AGW	7	100,1 %
2 AGW	7	96,8 %

Zusätzlich wurde die Möglichkeit einer Lagerung des Sammelmediums in Desorptionslösung geprüft. Dafür wurde die Sammelschicht der beladenen Röhrchen entnommen und, wie in Abschnitt 5.2 beschrieben, sofort mit Toluol überschichtet. Diese Lösungen wurden ohne weitere Aufbereitung für 1, 2 und 3 Wochen bei 1–5 °C gelagert und anschlie­ßend Aliquote entnommen und analysiert. Hierbei sank die Wiederfindung bereits nach 2 Wochen unter 80 %. Somit ist die Lagerfähigkeit auch mit diesem Vorgehen auf eine Woche beschränkt.

### 10.8 Störeinflüsse

**Abb.2:**
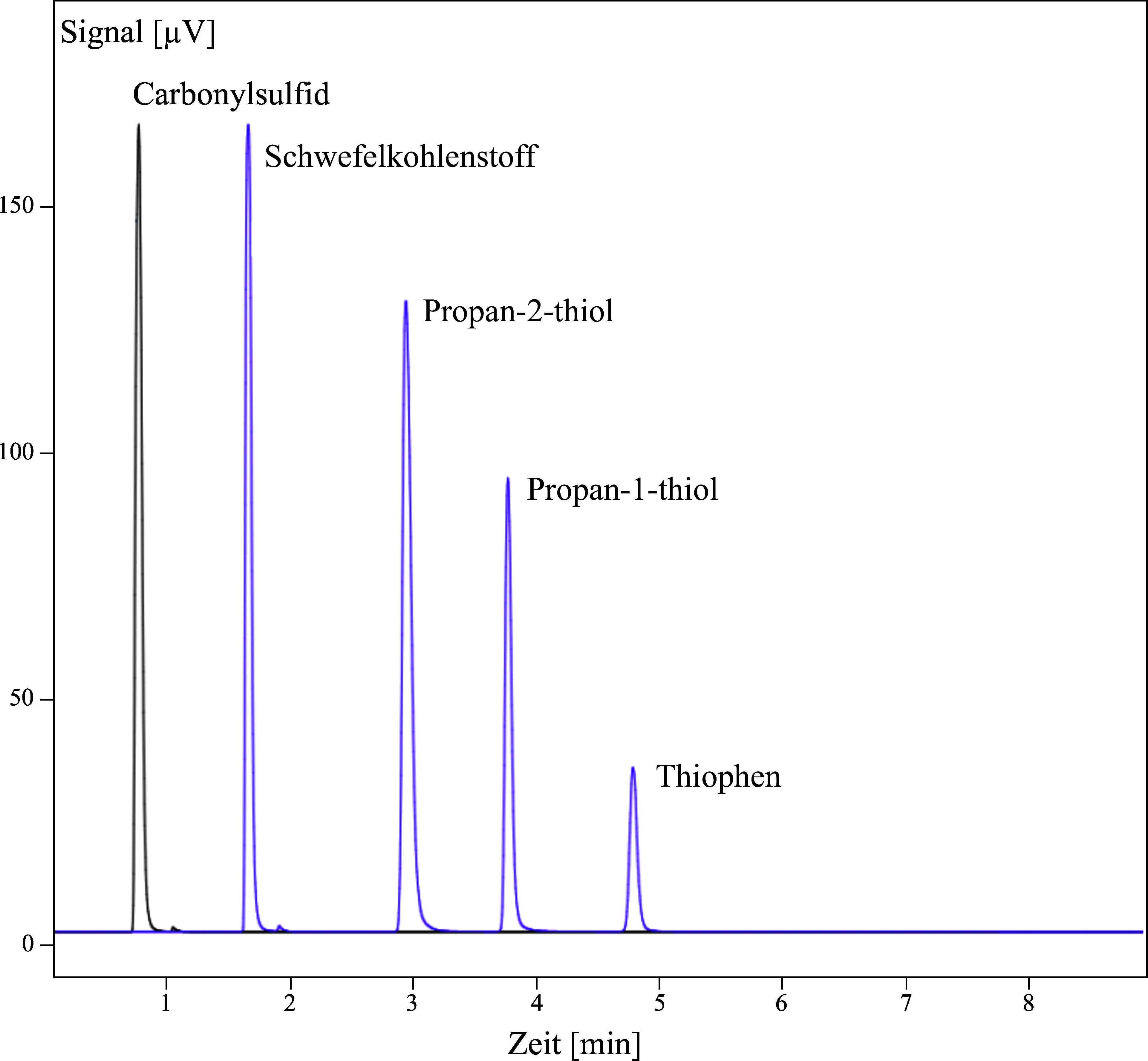
Übereinandergelegte Gaschromatogramme von Carbonylsulfid, Propan-2-thiol, Propan-1-thiol und Thiophen zur Überprüfung der Selektivität. Thiophenol ist unter den angegebenen chromatographischen Bedingungen nicht detektierbar

Mögliche Störeinflüsse wurden mit den Stoffen Carbonylsulfid, Propan-1-thiol, Propan-2-thiol und Thiophenol geprüft. Mit den Bedingungen unter Abschnitt 6 konnten keine Störeinflüsse festgestellt werden (siehe Abbildung 2).

### 10.9 Vergleichsexperimente

Zur Prüfung der Methode wurden Vergleichsexperimente mit einer unabhängigen Methode durchgeführt. Fünf Aktiv­kohleröhrchen wurden wie in Abschnitt 10 beschrieben für 120 min mit Prüfgas beaufschlagt und mit der hier vorgestellten Methode aufgearbeitet und analysiert (siehe Abschnitte 5.2, 6 und 7). Weitere fünf identisch beaufschlagte Aktivkohleröhrchen wurden unabhängig von der Fa. Analytik Service Obernburg GmbH aufgearbeitet und mit Hilfe einer photometrischen Methode analysiert. Die unabhängig voneinander ermittelten Wiederfindungen stimmen gut überein (siehe Tabelle 5).

**Tab.5:** Ergebnisse der Vergleichsexperimente

Methode	Sollkonzentration [mg/m^3^]	Ermittelte Konzentration [mg/m^3^]	Korrigierte mittlere Wiederfindung [%]
GC-FPD	30,86	29,80^a)^	96
Photometrie	30,86	31,46	102

a) Die gemessene Konzentration wurde mit einer mittleren Wiederfindung von 70 % korrigiert (siehe Abschnitt 10.2).

## 11 Diskussion

Das hier beschriebene Messverfahren ermöglicht die Bestimmung von Schwefelkohlenstoff in der Luft am Arbeitsplatz in einem Konzentrationsbereich von einem Zehntel bis zum Doppelten des derzeit gültigen AGW von 30 mg/m^3^. Die Bestimmungsgrenze ist darüber hinaus ausreichend, um den MAK-Wert von 16 mg/m^3^ als Beurteilungsmaßstab heranzuziehen. Die Methode wurde bei Temperaturen von 20 bis 30 °C validiert. Weichen die Bedingungen während der Messung signifikant davon ab, so ist das Verfahren entsprechend zu überprüfen. Die Methode ist geeignet, um die Einhaltung des Kurzzeitwertes zu überprüfen. Vergleichsmessungen mit einem unabhängigen Verfahren ergaben ähnliche Messwerte.
